# CD45RC Expression of Circulating CD8^+^ T Cells Predicts Acute Allograft Rejection: A Cohort Study of 128 Kidney Transplant Patients

**DOI:** 10.3390/jcm8081147

**Published:** 2019-08-01

**Authors:** Marie Lemerle, Anne-Sophie Garnier, Martin Planchais, Benoit Brilland, Yves Delneste, Jean-François Subra, Odile Blanchet, Simon Blanchard, Anne Croue, Agnès Duveau, Jean-François Augusto

**Affiliations:** 1 Service de Néphrologie-Dialyse-Transplantation, CHU Angers, 49000 Angers, France; 2 CRCINA, INSERM, Université de Nantes, Université d’Angers, 49100 Angers, France; 3 Service d’Immunologie et d’Allergologie, CHU Angers, 49000 Angers, France; 4 Centre de ressources biologiques, BB-0033-00038, Université d’Angers, CHU d’Angers, 49000 Angers, France; 5 Département de Pathologie Cellulaire et Tissulaire, CHU d’Angers, 49000 Angers, France

**Keywords:** kidney transplantation, acute rejection, lymphocyte, CD45RC

## Abstract

Predictive biomarkers of acute rejection (AR) are lacking. Pre-transplant expression of CD45RC on blood CD8^+^ T cells has been shown to predict AR in kidney transplant (KT) patients. The objective of the present study was to study CD45RC expression in a large cohort of KT recipients exposed to modern immunosuppressive regimens. CD45RC expression on T cells was analyzed in 128 KT patients, where 31 patients developed AR, of which 24 were found to be T-cell mediated (TCMR). Pre-transplant CD4^+^ and CD8^+^ CR45RC^high^ T cell proportions were significantly higher in patients with AR. The frequency of CD45RC^high^ T cells was significantly associated with age at transplantation but was not significantly different according to gender, history of transplantation, pre-transplant immunization, and de novo donor specific anti-Human Leucocyte Antigen (HLA) antibody. Survival-free AR was significantly better in patients with CD8^+^ CD45RC^high^ T cells below 58.4% (*p* = 0.0005), but not different according to CD4^+^ T cells (*p* = 0.073). According to multivariate analysis, CD8^+^ CD45RC^high^ T cells above 58.4% increased the risk of AR 4-fold (HR 3.96, *p* = 0.003). Thus, pre-transplant CD45RC expression on CD8^+^ T cells predicted AR, mainly TCMR, in KT patients under modern immunosuppressive therapies. We suggest that CD45RC expression should be evaluated in a prospective study to validate its usefulness to quantify the pre-transplant risk of AR.

## 1. Introduction

Significant progress has been made over the past few years in the immunological and histological fields, allowing for better differentiation and to refine the diagnosis and prognosis of T cell-mediated rejection (TCMR) and of antibody-mediated rejection (ABMR) in kidney transplant patients [1]. While both rejection types may develop concomitantly, TCMR mainly occurs within the first year post-transplant, while ABMR usually develops later in the course and is associated with the presence of preformed or de novo donor-specific anti-Human Leucocyte Antigen (HLA) antibodies (DSA) [1,2]. Although modern immunosuppressive regimens efficiently prevent allograft rejection in most patients, acute rejection (AR) episodes still occur in some patients and are associated with premature graft loss and morbidity [1,2,3].

Several risk factors of AR have been identified in previous studies including young age, female gender, black race, and immunological characteristics (HLA mismatch, pre-transplant or de novo DSA) [4]. However, despite being indicative at the population level, when considered at the individual level, most of these factors do not allow for the accurate stratification of AR risk, especially in “low immunological risk patients,” which represents most kidney transplant candidates. The identification of patients with higher versus lower AR risk among low immunological risk patients would theoretically allow one to tailor the immunosuppressive regimen according to the AR risk and thus to decrease post-transplant morbidity [5,6].

The risk of allograft rejection relies on graft-, environmental-, and host-related factors [1]. However, the molecular mechanisms underlining the development of alloreactivity are far from being fully understood [7]. An illustrative example of the inter-individual variability of AR risk is represented by operationally tolerant patients, defined as solid allograft recipients that do not develop allograft rejection despite immunosuppressive treatment discontinuation [7]. Thanks to intrinsic immunological factors, and probably also acquired factors, these patients are unable to mount an efficient alloreactive response.

The identification of biomarkers reflecting the level of tolerance emerges as a major goal in solid organ transplantation. This would allow one to tailor the immunosuppressive regimens, especially in low immunological risk kidney transplant candidates. Given that CD4^+^ and CD8^+^ T cell subsets have an essential role in the development of alloimmune response, defining T cell subpopulations with higher and lower alloimmune properties may constitute an interesting approach.

CD45 is a transmembrane protein tyrosine phosphatase heavily expressed on T cells and critical for signal transduction by regulating kinases of the Src-family [8,9]. Four CD45 isoforms (RO, RA, RB, RC), resulting from an alternative splicing of three exons, are expressed in humans [9]. The CD45RC isoform is highly expressed on human naive T cells with a bimodal and a trimodal pattern on CD4^+^ T cells (high and low expression) and CD8+ T cells (low, intermediate, and high) [10,11]. These patterns of expression define CD45RC T cell subsets with different cytokine profiles. Interestingly, the expression of CD45RC on T cells is highly variable between individuals and is genetically determined [10,11,12].

We demonstrated in a previous work that the level of CD45RC expression at the surface of blood CD8^+^ T cells before kidney transplantation was associated with the risk of AR after transplantation [10,13]. This study was conducted on a cohort of 89 kidney transplant recipients transplanted between 1999 and 2004, and we observed that a pre-transplant proportion of CD8^+^ CD45RC^high^ T cells above 54.7% conferred a 6-fold increased risk of developing AR after 4.8 years of follow-up [10]. The aim of the present study was thus to confirm this observation in a prospective cohort of kidney transplant patients treated with current immunosuppressive regimens.

## 2. Material and Methods

### 2.1. Study Design and Aim

This is a monocentric cohort study that included patients transplanted in the University Hospital of Angers between 2007 and 2015. During the period of the study, after giving their written consent, patients were offered the chance to participate to a biocollection (“Collection Néphrologie et voies urinaires”). Samples were collected before kidney transplantation and stored at the dedicated department (“Centre de Ressources Biologiques BB-0033-00038”). All patients that gave their written consent to the study were included. The primary aim of the study was to analyze the value of CD45RC expression on T cells for AR prediction. The study was approved by the Medical Ethics Committee of Angers University Hospital (2009/10).

### 2.2. Immunosuppressive Regimens

The immunosuppressive treatment was not imposed by the study and was based on the assessment of immunological risk according to clinical practice in our department as detailed hereafter. Low immunological risk patients, defined as first time kidney transplant recipients and with PRA < 20%, received two injections of Basiliximab (Simulect; Novartis Pharma, Basel, Switzerland), while higher immunological risk recipients (previous transplantation, PRA > 20%) were more likely to receive antithymocyte globulines (ATG; Thymoglobuline; Genzyme, Lyon, France) during the first 3 to 7 days post-transplant. ATG was also used for induction in donors with cardiac arrest before brain death, in non-heart-beating donors, and when delayed graft function was anticipated by clinician. Moreover, between 2010 and 2013, no induction therapy was performed in patients aged >70 years old. All patients received a single methylprednisolone bolus of 500 mg followed by prednisone (1 mg/kg/day) with a progressive tapering and discontinuation at the end of month 5 post-transplant, unless there was an occurrence of AR. A maintenance immunosuppressive regimen relied mainly on mycophenolate mofetil or mycophenolic acid and tacrolimus.

### 2.3. Data Collection and Definitions

Characteristics of the study population were collected prospectively via the systematic screening of patients’ medical records. All clinical events and biological data were retained until last follow-up: anthropometric data, nature of original kidney disease, and graft donor characteristics. Diagnosis of acute rejection (AR) episodes was based on conventional clinical and laboratory criteria and confirmed using a histological examination of a graft biopsy (according to the last Banff Classification) [14]. AR diagnosis was based on clinical and laboratory criteria (clinically diagnosed AR) when the graft biopsy was non-contributive or contra-indicated.

### 2.4. Sample Collection

Peripheral blood mononuclear cells (PBMC) of kidney transplant candidates were prospectively harvested before transplantation and stored in liquid nitrogen. Patients with samples showing PBMC viability below 80% were excluded from the analysis.

After giving their written consent, fresh samples of end-stage renal disease (ESRD) patients and heathy individuals (HD) were used to monitor the proliferation capacities of CD45RC T cells.

### 2.5. Antibodies and Flow Cytometry Analysis

The following conjugated antibodies were used to characterize CD45RC T cell subpopulations: CD3-VioGreen (REA613), CD4-PerCP-Vio700 (REA623), CD8-PE-Vio770 (REA734), from Milteny Biotec, Bergisch-Gladbach, Germany; CD45RA-APC (HI100) from BD Biosciences, San Jose, CA, USA; and CD45RC-FITC (MT2) from IQ Product, Houston, TX, USA. Cell viability was systematically assessed (LIVE/DEAD Fixable Near-IR Dead Cell Stain kit; Fischer Scientific, Pittsburgh, PA, USA). Briefly, 10^6^ cells were incubated with the viability dye according to the manufacturer’s recommendations before incubation with the antibodies. Data were collected using a FACS-Canto II (BD Biosciences) cytometer and analyzed using the FlowJo software, Ashland, OR, USA. The expression of CD45RC is bimodal on CD4^+^ T cells, some cells expressing low levels of CD45RC (CD45RC^low^), and others expressing high levels (CD45RC^high^). On CD8^+^ T cells, expression of CD45RC is trimodal, the first fraction of cells expressing low levels (CD45RC^low^), the second fraction expressing intermediate levels (CD45RC^Int^), and the last fraction expressing high levels of CD45RC (CD45RC^high^). Figure S1 illustrates the gating strategy.

### 2.6. CD45RC^+^ T Cell Purification and T Cell Proliferation Analyses

CD45RC T cells were sorted from freshly isolated PBMC of end-stage renal disease (ESRD) patients and age/sex matched healthy donors (HD) using a FACS-Aria cytometer, BD Bioscience, San Jose, CA, USA. Briefly, after a gradient centrifugation, 2 × 10^7^ PBMCs were stained using a Cell Trace Violet proliferation kit (Thermofischer, San Jose, CA, USA) for proliferation assessment and then stained using CD4-BV421 (L3T4, BD Biosciences), CD8-PE-Vio770 (REA734, Miltenyi Biotec), and CD45RC-FITC (MT2, IQ Product). CD45RC^high^ and CD45RC^low^ subpopulations were sorted among CD4^+^ and CD8^+^ T cells. Purity was always routinely above 95%. Then, 5 × 10^4^ T cells were cultured at 37 °C in RPMI 1640 medium (containing 8% fetal calf serum) in 96-well round-bottomed microplates (Becton Dickinson, Franklin Lakes, NJ, USA), with or without a 1 µg/mL plate-bound anti-CD3 (Beckman-Coulter, Brea, CA, USA) and 0.5 µg/mL soluble anti-CD28 (Beckman-Coulter). After 72 h of culture, cells were harvested and proliferation was assessed using flow cytometry (FACS-Canto II; BD Biosciences).

### 2.7. Statistical Analysis

Data were expressed as a median with minimum to maximum values for continuous variables and absolute count with percentage for categorical variables. Categorical and continuous data were analyzed with χ^2^ or Fischer’s exact test and Mann–Whitney U tests, respectively. The Wilcoxon matched-pairs rank test was used to compare the proliferative capacities of T cells. The predictive values of the CD45RC subset frequency for the first AR episode were analyzed using receiver operating characteristics (ROC) curves. Subsequently, cut-off values were determined by using the Youden index. The Kaplan–Meyer method was used to analyze AR-free survivals according to predetermined cut-off values of CD45RC subset frequencies. A log-rank test was used to compare survival curves. Correlations were analyzed using Spearman’s rank correlation test. Multivariate cox models were used to analyze the association between CD45RC subset frequencies and AR. Results are reported as hazard ratio (HR) with 95% CIs. All *p*-values were two-sided and a *p*-value lower than 0.05 was considered statistically significant. Statistical analysis was performed using Graphpad Prism^®^ version 7 (San Diego, CA, USA) and SPSS^®^ software version 22.0 (IBM, Armonk, NY, USA).

## 3. Results

### 3.1. Characteristics of the Population

**Figure 1:**
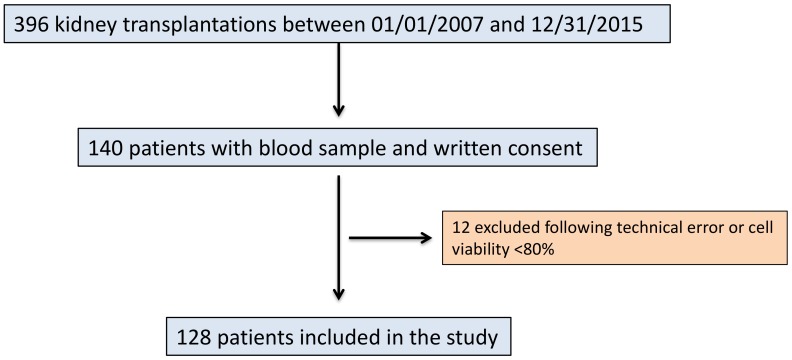
Flowchart of the study.

Between January 1, 2007, and December 31, 2015, 396 patients underwent kidney transplantation in Angers University hospital. Among them, 292 patients had blood samples collected and stored in a biocollection before transplantation, and 140 gave their written consent to participate in the present study. Among these 140 patients, samples from 12 patients were excluded because of technical errors (*n* = 6) or poor blood cell viability (*n* = 6). Thus, 128 patients were included and finally analyzed (Figure 1, flowchart).

The population was predominantly composed of males, with a median age of 50.2 years. The main cause of ESRD was autosomal dominant polycystic kidney disease and patients were first-time transplanted in 90% of cases. Based on PRA, 69.5% were non-sensitized before transplantation, while 6.25% of patients had a PRA > 20%. Basiliximab was used predominantly for induction in 56.3% of patients and most patients received tacrolimus with mycophenolate mofetil as a maintenance regimen. These data are detailed in Table 1.

### 3.2. Acute Rejection Episodes

The mean follow-up of the cohort was 3.82 ± 2.22 years. During the follow-up, AR occurred in 31 patients (24.2%) at a mean delay of 0.73 ± 1.24 years post-transplant. When considering only the first AR episode, 28 were histologically-proven and 3 were diagnosed based on clinical and biological criteria. Among the histologically-proven AR cases, 24 were TCMR, and 6 being borderlines. The four other AR episodes were ABMR in one case and mixed AR (TCMR and ABMR) in the three other cases. At one-year post-transplant, mean serum creatinine was 141.4 ± 75.2 µmol/L and mean glomerular filtration rate (GFR) was 53.2 ± 21.8 mL/min/1.73 m^2^. DSA developed in 15 patients (11.7%) during follow-up. These data are reported in Table 2.

Patients that experienced AR received more frequently Basiliximab as induction therapy as compared to patients that did not experienced AR, who received more-frequent ATG (*p* = 0.035). Baseline characteristics, including age and pre-transplant immunization, were not significantly different between groups. These data are reported in Table 3. When borderline ARs were excluded, no significant differences were observed between patients with and without AR (Table S1).

### 3.3. Proliferative Capacities of CD45RC T Cells

**Figure 2:**
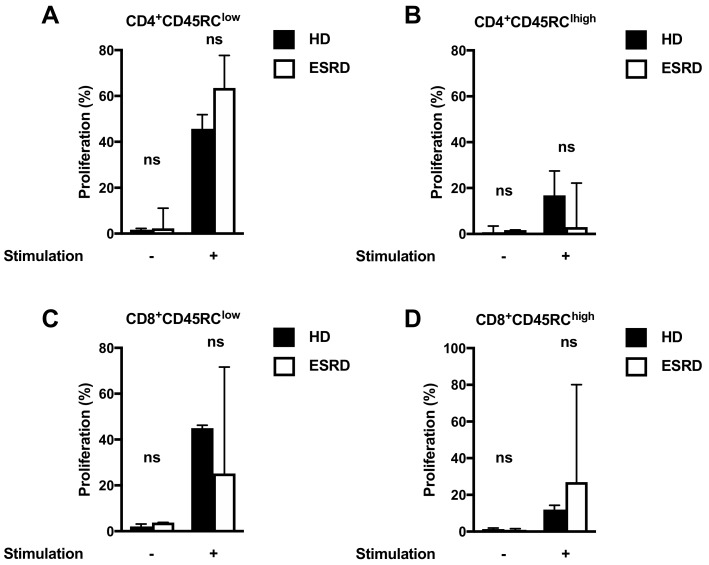
Analysis of proliferative capacities of CD45RC^low^ and CD45RC^high^ T cells in ESRD patients and HD. After 72 h, the proliferation of activated CD4^+^CD45RC^low^ (**A**), CD4^+^CD45RC^high^ (**B**), CD8^+^CD45RC^low^ (**C**), and CD8^+^CD45RC^high^ (**D**) T cell subsets of ESRD patients (black bars) and HD (white bars) was analyzed. The experiment reported results of four ESRD patients and four age and matched HD. Error bars show the median with a 95% CI. Comparisons were done using the Wilcoxon matched-pairs rank test. ns, non-significant. CI, Confidence Interval; ESRD: end-stage renal disease; HD: heathy individuals.

The proliferative properties of CD45RC T cells have been studied only in HD [10,11]. Thus, we analyzed the proliferative properties of subpopulations in ESRD patients as compared to age and sex-matched HD. As shown in Figure 2, proliferative properties of CD45RC T cells were not different between ESRD patients and HD, suggesting that their immune function was maintained in ESRD.

### 3.4. Association between CD45RC Expression on T Cells and Acute Rejection

**Figure 3:**
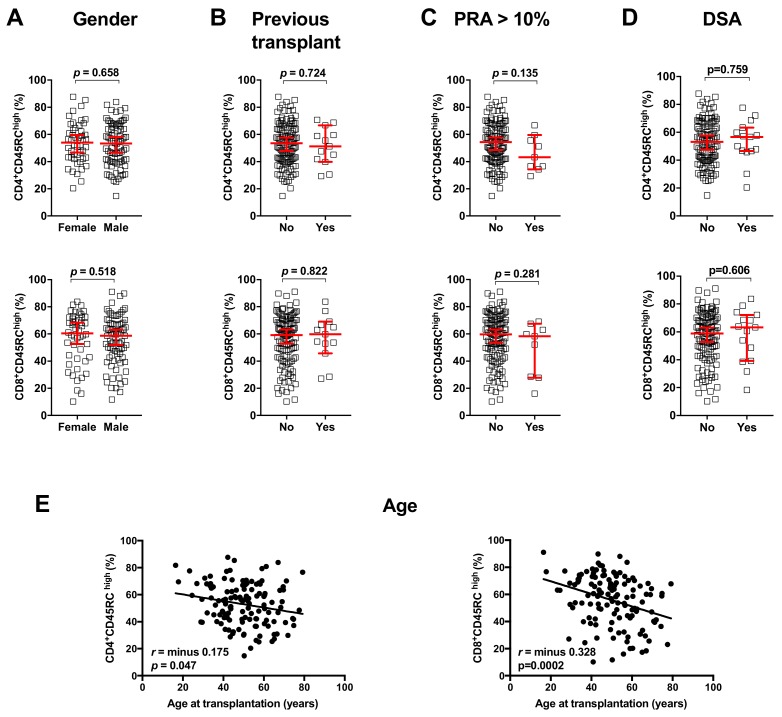
Proportion of CD45RC^high^ and CD45RC^low^ CD4+ and CD8+ T cells according to gender (**A**), previous transplantation (**B**), pre-transplant PRA (**C**), de novo DSA occurrence (**D**), and age at transplantation (**E**). For A–D, comparisons were done using the Mann–Whitney U test and error bars show median with a 95% CI. For E, correlation analysis was done using the Spearman test. CI, Confidence Interval; PRA, Panel Reactive Antibody.

We first analyzed the association of CD45RC expression on T cells with patient’s characteristics. As shown in Figure 3, the level of CD45RC expression on CD4^+^ and CD8^+^ T cells were not significantly different according to gender, the number of previous transplantations, the level of pre-transplant immunization, or de novo DSA development. As previously reported in healthy subjects [10,11], CD45RC expression on CD4^+^ and CD8^+^ T cells was correlated with age (*p* = 0.047 and *p* = 0.0002, respectively).

We next analyzed the frequency of CD45RC T cell subsets according to the occurrence of AR (Table 4). In line with our previous observations [10], patients who experienced AR had a higher proportion of CD4^+^ and CD8^+^ CD45RC^high^ compared to patients that did not develop AR. The difference between groups remained significant regardless of whether all AR episodes were considered, when analysis was restricted to biopsy-proven ARs, or when borderline AR episodes were excluded. Moreover, the absolute number of both CD4^+^ and CD8^+^ CD45RC^high^ cells was significantly greater in patients that experienced AR (*p* = 0.0101 and 0.0073, respectively; Figure S2).

### 3.5. Value of CD45RC Expression on T Cell for Acute Rejection Prediction

**Figure 4:**
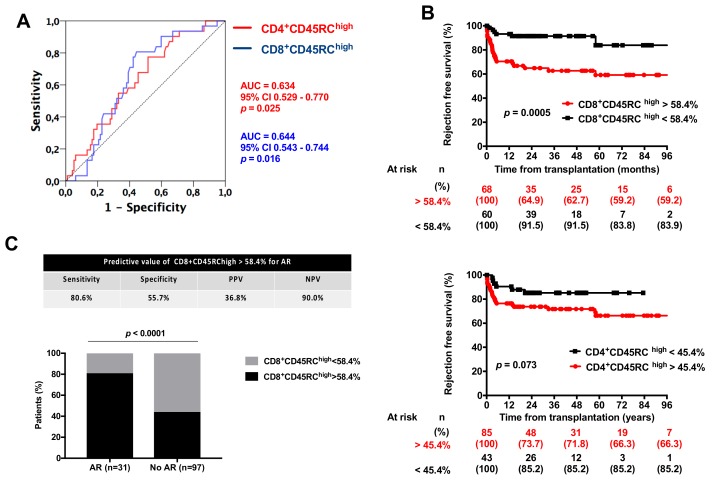
Predictive value of CD4^+^ and CD8^+^ CD45RC T cell subsets. (**A**) ROC curve analysis of CD4^+^ and CD8^+^ CD45RC^high^ T cell subsets for AR prediction. (**B**) Rejection-free survival of patients according to CD8^+^ (upper panel) and CD4^+^ CD45RC^high^ T cell proportions. Comparison between survivals was done using a log-rank test. (**C**) predictive values of CD8^+^ CD45RC^high^ T cell above 58.4% for AR prediction. AUC: Area Under Curve.

We next analyzed the best thresholds of CD4^+^ and CD8^+^ CD45RC^high^ T cell frequencies for AR prediction. Using ROC curve analysis, we could determine 45.4% and 58.4% as the best thresholds of CD4^+^ and CD8^+^ CD45RC^high^ frequencies for AR prediction, respectively (Figure 4A). Using these thresholds, we observed that AR-free survival was significantly greater in patients with a CD8^+^ CD45RC^high^ T cell frequency below 58.4% (*p* = 0.0005), while AR-free survival was not significantly different according to CD4^+^ CD45RC^high^ T cell frequencies (*p* = 0.073) (Figure 4, upper and lower panel). Thus, CD8^+^ CD45RC^high^ T cell frequency allows one to better differentiate patients at risk of AR, as compared to CD4^+^ CD45RC^high^ T cells. The sensitivity, the specificity, and the positive predictive and negative predictive values of CD8^+^ CD45RC^high^ T cell frequency above 58.4% for ARs were 80.6%, 55.7%, 36.8%, and 90%, respectively. These results are illustrated in Figure 4C. We did not observe any correlation between TCMR severity and the proportion of CD8^+^ CD45RC^high^ T cells (Figure S3).

We finally analyzed the risk factors of AR in a cox model analysis (Table 5). We successively considered all ARs (including those clinically diagnosed), ARs excluding borderline cases, and biopsy-proven ARs. A CD8^+^ CD45RC^high^ T cell frequency above 58.4% was significantly associated with AR after adjustment on type of induction. The CD8^+^ CD45RC^high^ T cell frequency and ATG as an induction treatment were both associated with AR (all, including borderline) and biopsy-proven AR. When borderline ARs were excluded, ATG was no longer significantly associated with AR occurrence.

## 4. Discussion

In the present work, we confirmed that the CD8^+^ CD45RC^high^ T cell subset was associated with an increased risk of AR. We could determine that patients with a proportion of CD8^+^ CD45RC^high^ T cell frequency above 58.4% had a 4-fold increased risk of AR. Thus, these results are in line with our previous work [10,13] and confirm the interest of CD45RC expression on T cells to assess the immunological risk of candidates to kidney transplantation.

As compared to our previous work [10,13], in the present study, patients were treated with current immunosuppressive strategies. Indeed, most patients received Basiliximab as an induction regimen and tacrolimus maintenance, while ATG and tacrolimus monotherapy after 6 months post-transplant was the most commonly used regimen in our previous study. Another important difference is that patients considered at high immunological risk were included in the present study. Although, pre-transplant sensitized patients represented a minority of the study population, we suggest that CD45RC expression may also help determine the AR risk in this specific population. Interestingly, CD45RC expression was not significantly different between patients with or without previous kidney transplantation or who were sensitized before kidney transplantation, which suggests that previous exposure to immunosuppressive drugs may not affect CD45RC expression on T cells. As previously reported, CD45RC expression was negatively associated with age, and correlation was greater for the CD8^+^ T cell subset [10,11]. A genetic control of CD45RC on T cells has been demonstrated in rats [12,15]; however, its regulation in humans remains largely unknown.

AR incidence, including borderline AR, in our study was 24.2% after a mean follow-up of almost 4 years. As expected, most AR episodes were TCMR, with ABMR being implicated in only four AR episodes. This observation is in line with recent reports [3,16] showing the predominant occurrence of TCMR as compared to ABMR in the first few years of transplantation. Most AR episodes occurred within the first few years following kidney transplantation, which is also in line with published data [17,18]. Thus, CD45RC expression mainly allows one to assess the risk of TCMR, which represented the majority of ARs in the cohort. Interestingly, we did not observe any difference in CD45RC^high^ T cell subset frequency according to subsequent DSA development, which suggests that CD45RC status does not allow one to assess the risk of ABMR. TCMR still represents a significant cause of morbidity in the era of ABMR, the latter being the main cause of late graft lost [19]. Moreover, several recent reports suggested that the occurrence of TCMR is independently linked to subsequent interstitial fibrosis development [20] and probably an increased risk of ABMR development by exposing HLA antigens within kidney [18,21,22]. Thus, these data suggest that decreasing the rate of TCMR, including sub-clinical TCMR, could allow one to improve the long-term graft outcome and decrease the risk of ABMR. This reinforces the need to better delineate patients at high TCMR risk to prevent it by adjusting the immunosuppressive regimen.

Interestingly, not only CD45RC proportion was associated with AR occurrence, but also the absolute count of CD45RC^high^ CD4^+^ and especially CD8^+^ T cells. In vitro, CD45RC^high^ CD8^+^ T cells from HD mainly produced interferon gamma a key cytokine in TCMR, and poor levels of regulatory cytokines [10]. Whether, the ESRD milieu affects the cytokine profile of CD45RC T cell subsets remains to be fully analyzed. However, we show here that CD45RC T cell subsets from ESRD patients had a similar proliferative capacity as compared to those of HD. This suggests that the immune functions of CD45RC T cell subsets were preserved in a uremic context.

We could determine in the present study that 58.4% was the best threshold of CD8^+^ CD45RC^high^ T cells for AR prediction. In our previous work, the best threshold was determined at 54.7% [10]. Finally, moving the cut-off to this value would result in minimal changes in the predictive value of CD8^+^ CD45RC^high^ T cells in the present study. The very closed cut-off values as determined in the two works, conducted in two different populations with different immunosuppressive regimens, reinforces the strength of this biomarker.

The present study has several limitations. First, only 32% of patients transplanted during the period were included and we could not exclude a selection bias. Next, AR diagnosis was based on for-cause biopsies and not protocol biopsies. Thus, the value of CD45RC expression for subclinical rejection remains to be investigated. Moreover, given the relatively short follow-up, we were not able to analyze the relationship between pre-transplant CD45RC expression and the risk of ABMR, which occurred in only four patients in the present study.

In conclusion, the present study confirmed and extended the data on CD45RC that has appeared as a promising biomarker to assess the risk of AR before transplantation. This work confirmed that the pre-transplant proportion of CD8^+^ CD45RC^high^ T cells is associated with a 4-fold increased risk of AR, mainly TCMR. Thus, CD45RC could be used to help define the immunological risk and the level of immunosuppressive regimen before kidney transplantation. However, the value of CD45RC expression on T cells should be evaluated in a multicenter prospective cohort study with protocol biopsies to confirm its usefulness and to study its predictive value for subclinical AR.

## Figures and Tables

**Table 1:** Baseline characteristics of the population. Results are presented as a median with minimum to maximum value ranges for continuous variables and absolute count and percentage for categorical variables.

	All Patients
	(*n* = 128)
**Baseline Characteristics**	
Sex (M/F)	80/48
Age (years)	50.2 (18.0–79.2)
Weigh (kg)	71.0 (41.0–115.0)
BMI (kg/m^2^)	25.0 (17.3–40.2)
**Original nephropathy, *n* (%)**	
ADPKD	33 (25.8)
IgA nephropathy	17 (13.3)
Other GN	13 (10.2)
TIN/urologic	13 (10.2)
Vascular nephropathy/diabetic GN	13 (10.2)
Vasculitis	3 (2.3)
Lupus nephritis	5 (3.9)
Undetermined nephropathy	18 (14.1)
Others	13 (10.2)
**History of transplantation**	
Pre-transplant dialysis, n (%)	92 (71.9)
Previous kidney transplantation, n (%)	13 (10.2)
Donor age, years	50.0 (3.0–87.0)
Cold ischemia time (hours)	16.6 (2.0–35.4)
HLA mismatch	
HLA A&B&DR	4.0 (0–6)
HLA A&B	3.0 (0–4)
HLA DR	1.0 (0–2)
Sensitization, *n* (%)	
Nonsensitized at transplantation	89 (69.5)
PRA < 10%	30 (23.4)
PRA 10–20%	1 (0.8)
PRA > 20%	8 (6.2)
**Immunosuppressive regimens**	
Induction therapy	
None, *n* (%)	6 (4.7)
Basiliximab, *n* (%)	72 (56.3)
Antithymocyte globulins, *n* (%)	50 (39.1)
Maintenance regimen	
Tac-based, *n* (%)	102 (79.6)
Cyclosporin-based, *n* (%)	26 (20.3)
MMF or MPA, *n* (%)	127 (99.2)

ADPKD, autosomic dominant polycystic kidney disease; BMI, body mass index; GN, glomerulonephritis; HLA, human leukocyte antigens; MMF, mycophenolate mofetil; MPA, mycophenolic acid; PRA, Panel Reactive Antibody; Tac, tacrolimus; TIN, tubulo-interstitial nephropathy.

**Table 2:** Acute rejection episodes. Results are presented as a median with minimum to maximum value ranges for continuous variables and absolute count and percentage for categorical variables.

**Mean Follow-Up (Years)**	3.82 ± 2.22 (0.02–8.53)
**Acute Rejection**	
Number of patients, *n* (%)	31 (24.2)
Mean delay to first AR (years)	0.73 ± 1.24 (0.02–4.83)
Histologically proven, *n* (%)	28 (90.3)
TCMR	24 (85.7)
Borderline	6 (25.0)
Grade IA	9 (37.5)
Grade IB	8 (33.3)
Grade IIA	1 (4.2)
AMR	1 (3.6)
Mixed AR	3 (10.7)
Non histologically proved AR	3 (9.7)
More than one AR episode	9 (7.0)
**DSA, *n* (%)**	15 (11.7)
Class I	4 (26.7)
Class II	11 (73.3)
**Year 1 Post-Transplant Biological Results**	
Serum creatinine (µmol/L) *	141.4 ± 75.2 (60.0–716)
GFR (mL/min/1.73 m^2^) *	53.2 ± 21.8 (7.3–123)
Proteinuria/Creatininuria (g/g) *	0.25 ± 0.69 (0–5.78)

* In patients followed at the indicated time; AR, acute rejection; DSA, donor specific antibodies; GFR, glomerular filtration rate; disorder; TCMR, T-cell-mediated rejection; AMR, antibody-mediated rejection.

**Table 3:** Univariate analysis of factors associated with acute rejection occurrence. Comparisons between groups were done using the Mann–Whitney U test for continuous variables and Χ^2^ or Fisher exact tests for categorical variables. Continuous variables are expressed as a median with minimum to maximum values and categorical variables are expressed as their absolute count and percentage. Acute rejection groups were compared to the No AR group. Significant *p*-values appear in bold.

	No AR (*n* = 97)	AR (*n* = 31)	*p*	BPAR (*n* = 28)	*p*	Excluding Bor AR (*n* = 22)	*p*
**Baseline Characteristics**							
Sex (M/F)	62/35	18/13	0.558	16/12	0.514	12/10	0.413
Age (years)	50.3 (18.0–79.2)	48.2 (23.3–66.5)	0.301	48.8 (23.3–66.5)	0.368	45.5 (23.3–66.5)	0.206
No pretransplant immunization, *n* (%)	64 (66.0)	25 (80.6)	0.123	23 (82.1)	0.160	18 (81.8)	0.203
PRA > 20%, *n* (%)	7 (7.2)	1 (3.2)	0.679	1 (3.6)	0.682	1 (4.5)	1.000
**History of Transplantation**							
Previous kidney transplantation, *n* (%)	11 (11.3)	2 (6.5)	0.733	2 (7.1)	0.731	2 (9.1)	1.000
Pre-transplant dialysis, *n* (%)	77 (72.2)	22 (71.0)	0.897	20 (71.4)	0,939	15 (68.2)	0.709
Donor age, years	51.0 (3.0–87.0)	47.0 (36.0–80.0)	0.799	48 (36.0–80.0)	0.769	43 (37.0–68.0)	0.494
Cold ischemia time (hours)	17.0 (2.0–35.4)	15.6 (2.0–32.2)	0.434	15.5 (2.0–32.2)	0.299	15.5 (2.0–32.2)	0.359
HLA mismatch (ABDR), *n*	4.0 (0–6)	4.0 (0–6)	0.733	4.0 (0–6)	0.495	4.0 (0–6)	0.750
Delayed graft function, *n* (%)	22 (22.7)	6 (19.4)	0.658	5 (17.9)	0.551	4 (18.2)	0.779
De novo DSA, *n* (%)	12 (12.4)	3 (9.7)	1.000	3 (10.7)	1.000	3 (13.6)	1.000
**Immunosuppressive Regimens**							
Induction (none/Basiliximab/ATG), *n* (%)	4 (4.1)/49 (50.5)/44 (45.4)	2 (6.5)/23 (74.2)/6 (19.3)	**0.035**	2(7.1)/21(75.0)/5(17.9)	**0.031**	1(4.6)/16(72.7)/5(22.7)	0.145
Tacrolimus-based regimen, *n* (%)	78 (80.4)	23 (74.2)	0.393	22 (78.6)	0.752	16 (72.7)	0.370

AR, acute rejection; BPAR, biopsy proven acute rejection; Bor AR, borderline AR.

**Table 4:** Frequency of CD4^+^ and CD8^+^ CD45RC subsets according to AR occurrence. Comparisons were done using the Mann–Whitney U test. Significant *p*-values appear in bold.

Acute Rejection (all)	Yes *n* = 31	No *n* = 97	*p*
CD4^+^CD45RC ^high^	58.4 ± 13.7	51.2 ± 15.7	**0.023**
CD8^+^CD45RC ^high^	62.5 ± 13.3	53.6 ± 19.3	**0.019**
CD8^+^CD45RC ^int^	20.1 ± 7.7	23.2 ± 9.3	0.096
CD8^+^CD45RC ^low^	17.9 ± 9.8	23.7 ± 14.2	**0.035**
Biopsy-Proven AR *	**Yes** ***n* = 28**	**No** ***n* = 97**	***p***
CD4^+^CD45RC ^high^	59.2 ± 13.3	51.2 ± 15.7	**0.016**
CD8^+^CD45RC ^high^	62.3 ± 13.0	53.6 ± 18.0	**0.010**
CD8^+^CD45RC ^int^	20.1 ± 7.9	23.2 ± 9.3	0.117
CD8^+^CD45RC ^low^	18.0 ± 10.3	23.7 ± 14.2	**0.049**
Acute Rejection (excluding borderline AR) **	**Yes** ***n* = 22**	**No** ***n* = 97**	***p***
CD4^+^CD45RC ^high^	60.0 ± 13.4	51.2 ± 15.7	0.016
CD8^+^CD45RC ^high^	64.4 ± 12.2	53.6 ± 19.3	0.014
CD8^+^CD45RC ^int^	19.7 ± 8.1	23.2 ± 9.3	0.110
CD8^+^CD45RC ^low^	16.4 ± 7.9	23.7 ± 14.2	0.020

* Patients with clinical diagnosed AR without biopsy were excluded. ** Patients with biopsy-proven AR excluding patients with borderline AR. CD45RC subsets were determined as specified in the Materials and Method section. High, high expression; Low, low expression; Int, intermediate expression.

**Table 5:** Multivariate cox analysis of factors associated with acute rejection occurrence. Significant *p*-values appear in bold.

	Multivariate Cox Models	HR	95% CI	*p*
**All ARs**	CD8^+^CD45RC^high^ (>58.4%)	4.04	1.65–9.88	**0.002**
	Induction (ATG)	0.39	0.16–0.94	**0.037**
ARs excluding borderlines *	CD8^+^CD45RC^high^ (>58.4%)	4.42	1.49–13.1	**0.007**
	Induction (ATG)	0.46	0.17–1.25	0.130
Biopsy-proven ARs **	CD8^+^CD45RC^high^ (>58.4%)	3.59	1.45–8.89	**0.006**
	Induction (ATG)	0.35	0.13–0.93	**0.035**

* Patients with a biopsy–proven AR excluding patients with a borderline AR. ** Patients with a clinically diagnosed AR without biopsy were excluded.

## Supplementary Materials

The following are available online at https://www.mdpi.com/2077-0383/8/8/1147/s1. Figure S1. Representative experiment showing the cytometry gating strategy. Figure S2. Absolute number of CD4^+^ and CD8^+^ CDR45RC^high^ T cells according to AR occurrence. Figure S3. Correlation between CD8^+^ CD45RC^high^ T cell proportion and the severity of TCMR. The correlation was analyzed using Spearman’s test. Table S1: Univariate analysis of factors associated with acute rejection occurrence. Patients with borderline AR were excluded from the analysis.

## Abbreviations

ABMR	antibody-mediated rejection
AR	acute rejection
ATG	anti-thymocytes globulins
DSA	Donor specific anti-HLA antibodies
HD	healthy donors
PRA	panel reactive antibody
PBMC	plasma blood mononuclear cells
ROC	receiver operating curve
TCMR	T-cell-mediated rejection
