# Supplementary material for: CD45RC Expression of Circulating CD8+ T Cells Predicts Acute Allograft Rejection: A Cohort Study of 128 Kidney Transplant Patients
Source: J Clin Med. 2019 Aug 1;8(8):1147. doi: 10.3390/jcm8081147 (PMC6723395; doi:10.3390/jcm8081147)
Supplement: Supplementary file 1 [file jcm-08-01147-s001.pdf]

S1 Table. Univariate analysis of factors associated with acute rejection occurrence, after exclusion of borderline AR. Comparisons between groups were done using Mann-Whitney U test for continuous variables and Khi2 or Fisher exact tests for categorical variables. Continuous variables are expressed by median with minimum to maximum values and categorical variables are expressed by their absolute count and percentage.

	**Acute rejection (n=22)**	**No rejection (n=97)***	***p***
**Baseline characteristics**			
Sex (M/F, n (%))	12 (54.5)/10 (45.5)	62 (63.9)/35 (36.1)	0.413
Age (years)	46.6 ± 10.7	50.8 ± 13.3	0.170
**History of transplantation**			
Previous kidney transplantation, n (%)	2 (9.1)	11 (11.3)	1.000
Pre-transplant dialysis, n (%)	15 (68.2)	70 (72.2)	0.709
Donor age, years	50.1 ± 13.3	51.0 ± 15.6	0.792
Cold ischemia time (hours)	15.4 ± 6.4	17.1 ± 7.0	0.361
HLA mismatch (ABDR), n	3.77 ± 1.3	3.78 ± 1.3	0.974
**Immunosuppressive regimens**			
Induction (none/basiliximab/ATG), n (%)	1 (4.5)/16 (72.7)/5 (22.7)	4 (4.1)/49 (50.5)/44 (45.4)	0.145
Tacrolimus-based regimen, n (%)	16 (72.7)	78 (80.4)	0.370

* Patients with borderline AR were excluded from analysis.

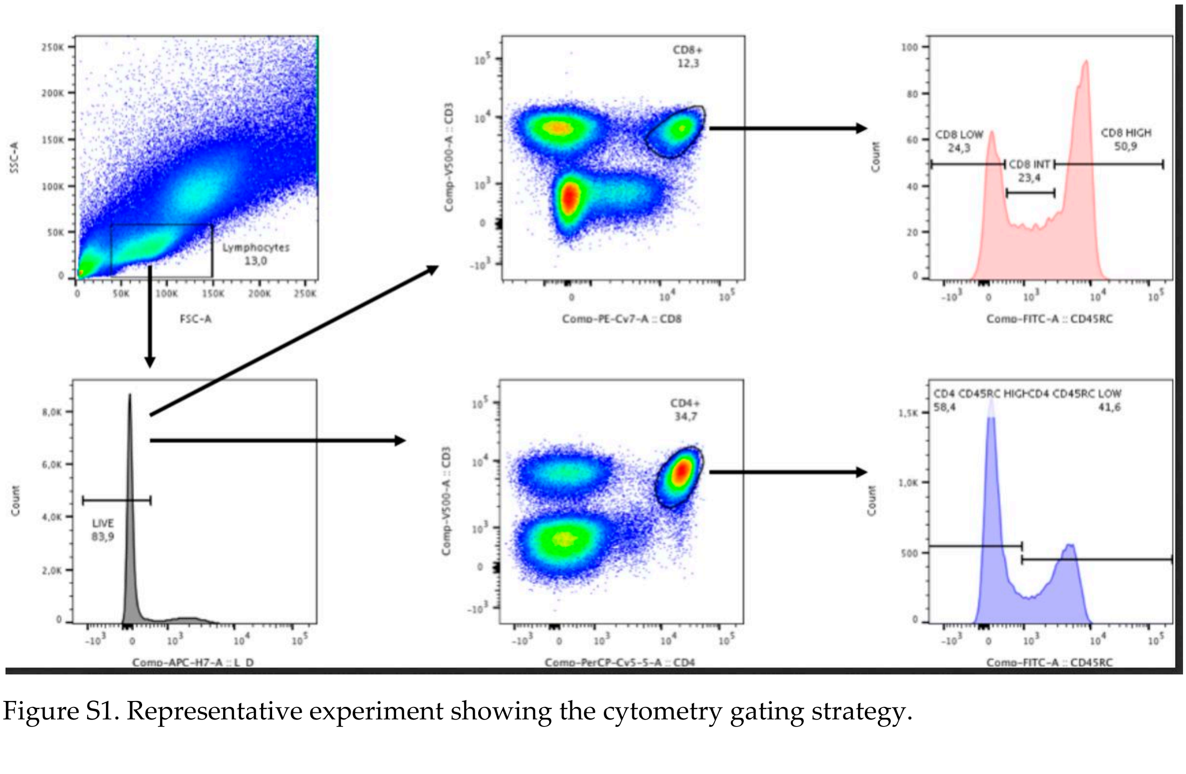

Figure S1. Representative experiment showing the cytometry gating strategy.

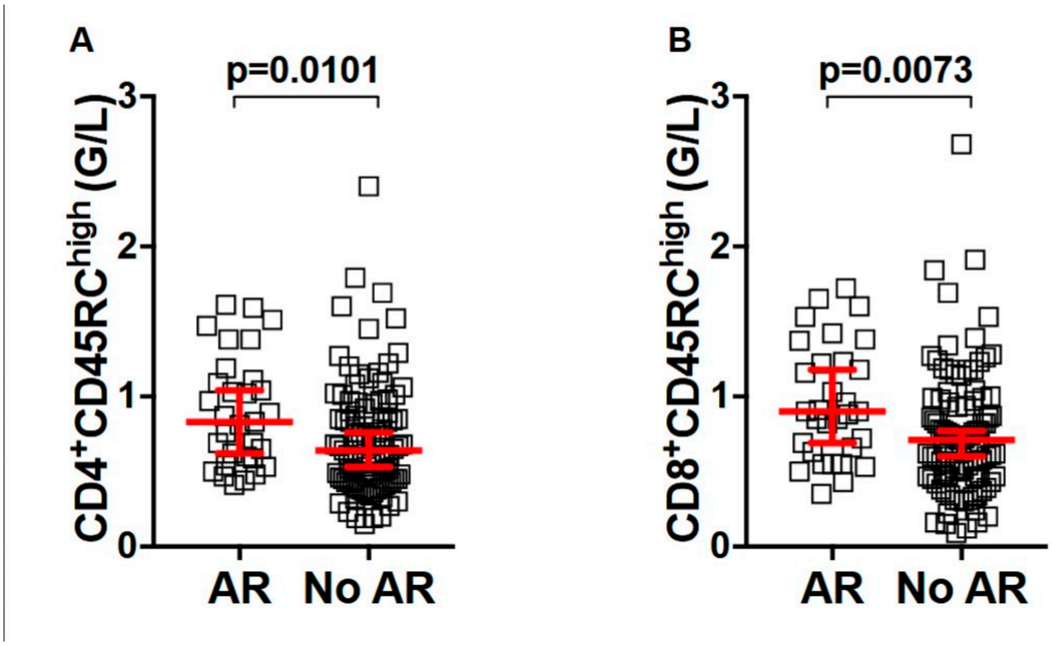

Figure S2. Absolute number of $CD4^{+}$ and $CD8^{+}$ $CDR45RC^{high}$ T cells according to AR occurrence.

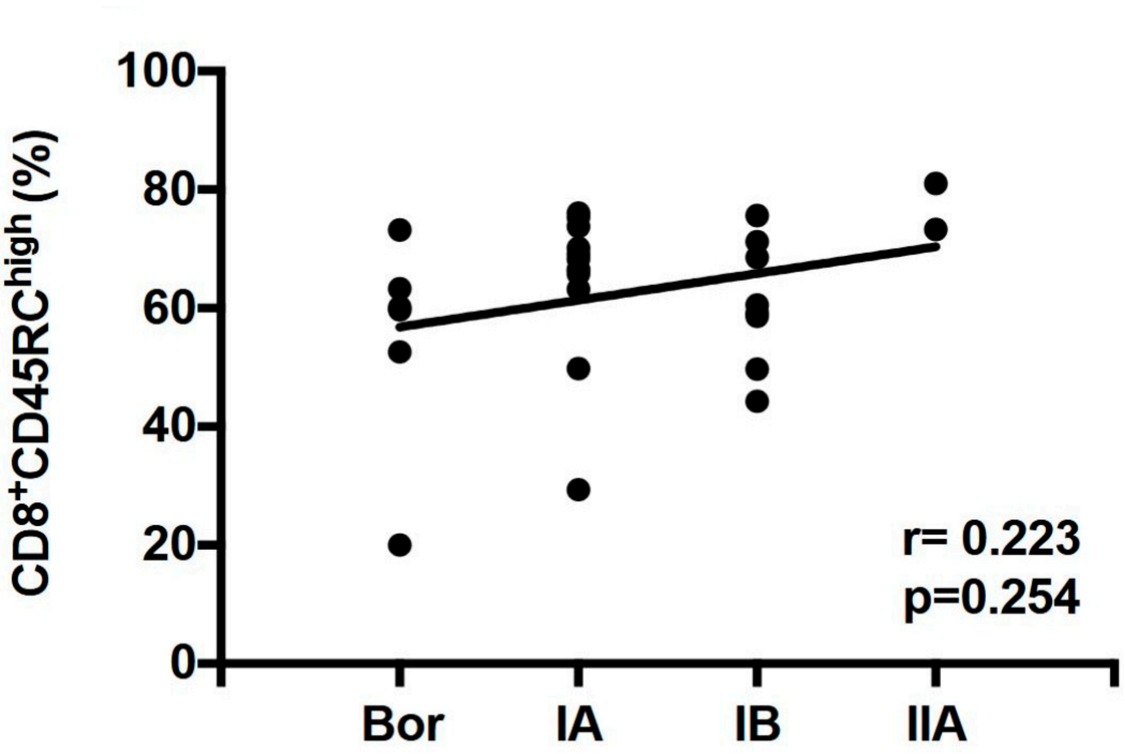

Figure S3. Correlation between $CD8^{+}$ $CD45RC^{high}$ T cell proportion and the severity of TCMR. The correlation was analyzed using Spearman's test.
